# Effect of Chitooligosaccharides‐Combination Fortified Noodles Intervention on Visceral Adiposity and Serum Metabolites in a High‐Risk Occupational Cohort of Young Seafarers

**DOI:** 10.1002/fsn3.72101

**Published:** 2026-07-25

**Authors:** Zhongbo Bian, Jiaming Wang, Yi Lu, YanYing Yao, Xiangru Feng, Xinjie Zhou, Xiao guo Ji, Juan Li

**Affiliations:** ^1^ Department of Nutrition, Shanghai Changzheng Hospital Naval Medical University Shanghai China; ^2^ School of Public Health Shanghai University of Traditional Chinese Medicine Shanghai China; ^3^ State Key Laboratory of Bioreactor Engineering East China University of Science and Technology Shanghai China

**Keywords:** chitooligosaccharides, metabolomics, seafarers, visceral adiposity

## Abstract

Offshore seafarers face a unique “metabolically obesogenic” environment, predisposing them to an elevated risk of central obesity and metabolic disorders, even at a young age, creating a critical need for feasible, on‐board nutritional interventions. This study aimed to evaluate the effects of Chitooligosaccharides‐combination fortified noodles on body composition in young male seafarers and to elucidate the underlying systemic metabolic mechanisms. A self‐controlled trial was conducted with 73 participants, who underwent a 4‐week placebo phase followed, after a washout, by a 4‐week intervention phase consuming functional noodles every other day. Clinical assessments included body weight, waist circumference, body composition indices, and serum lipid profiles, and untargeted metabolomics was performed on a subset of 38 participants. Compared with the placebo phase, oligosaccharide intervention significantly reduced waist circumference (WC), visceral fat area (VFA), serum leptin and LDL‐cholesterol (LDL‐C) levels, and increased skeletal muscle mass index, with no significant change in body weight. Linear mixed models confirmed significant net reductions in VFA (*β* = −18.36 cm^2^, 95% CI: −31.56 to −5.16; *p* < 0.05), WC (*β* = −7.51 cm, 95% CI: −10.78 to −4.23; *p* < 0.001), and LDL‐C (*β* = −0.33 mmol/L, 95% CI: −0.55 to −0.11; *p* < 0.005) following the intervention. Untargeted metabolomics revealed profound perturbations in the serum metabolome, with glycerophospholipid and thiamine metabolism identified as the most significantly dysregulated pathways. Key metabolites in these pathways exhibited marked alterations: phosphatidylethanolamine PE(18:3/12:0) and thiamine triphosphate (ThTP) were upregulated, while lysophosphatidylserine LysoPS(19:0/0:0) was downregulated. These metabolic changes were strongly associated with clinical improvements, with elevations in PE(18:3/12:0) and ThTP being inversely correlated with reductions in VFA, WC, and LDL‐C, and the decrease in LysoPS(19:0/0:0) positively correlated with these same clinical changes. Subgroup analysis indicated that seafarers with elevated central adiposity at baseline had a deficient metabolic state with lower levels of PE(18:3/12:0) and ThTP, and these participants derived greater corrective benefits in these metabolites and VFA reduction from the intervention; notably, baseline LysoPS(19:0/0:0) levels were comparable between subgroups, but its downregulation in response to the intervention was more pronounced in non‐centrally obese individuals. A four‐week dietary intervention with Chitooligosaccharides‐combination fortified noodles effectively reduced visceral adiposity and improved body composition in young seafarers, with benefits associated with targeted remodeling of glycerophospholipid and thiamine metabolism. The greater corrective response in individuals with pre‐existing central obesity highlights the potential of this strategy for precision nutrition in high‐risk occupational groups.

## Graphical abstract

In a 4‐week self‐controlled trial of 73 young seafarers, consumption of chitooligosaccharides‐combination fortified noodles every other day significantly reduced visceral fat area, waist circumference, serum leptin, and LDL‐C, and elevated skeletal muscle index, without altering body weight. Metabolomic analysis revealed remodeling of glycerophospholipid and thiamine pathways, with key differential metabolites closely correlated with improvements in adiposity and lipid profiles. Subgroup analysis demonstrated a more pronounced metabolic corrective response in participants with central obesity.

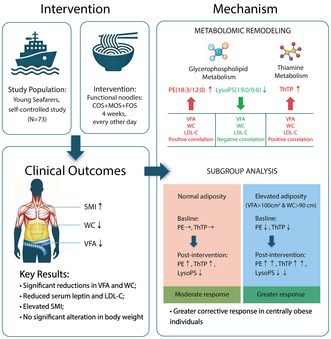

**Abbreviations**
AMC: upper arm muscle circumference
BIA: bioelectrical impedance analysis
BMC: bone mineral content
FDR: False Discovery Rate
HALLA: Hidden Analysis of Latent Variable Associations
HDL‐C: HDL‐cholesterol
KEGG: Kyoto Encyclopedia of Genes and Genomes
LC/MS: liquid chromatography‐mass spectrometry
LDL‐C: LDL‐cholesterol
LysoPS: lysophosphatidylserine
OPLS‐DA: Orthogonal Partial Least Squares Discriminant Analysis
PE: phosphatidylethanolamine
PLS‐DA: Partial Least Squares‐Discriminant Analysis
SMI: skeletal muscle mass index
SMM: skeletal muscle mass
TG: triglycerides
ThTP: thiamine triphosphate
VFA: visceral fat area
WC: waist circumference

## 1 Introduction

Metabolic syndrome and its associated conditions, including central obesity, dyslipidemia, and insulin resistance, represent a significant and growing global health burden. Certain occupational groups face a disproportionately high risk due to the unique interplay of environmental, dietary, and lifestyle factors inherent to their work. Offshore seafarers are a prime example, operating within a “metabolically obesogenic” environment characterized by prolonged periods of confined duty, shift work, psychological stress, and a persistent lack of access to fresh, nutrient‐dense foods (Russo et al. 2023; Nittari et al. 2019; Baygi et al. 2022). Their diets consequently rely heavily on energy‐dense, highly processed staples, which can lead to deficiencies in key micronutrients and dietary fiber (Oldenburg et al. 2013). This exposure profile contributes to a high prevalence of central obesity, unfavorable lipid profiles, and elevated cardiovascular disease risk in this population. Implementing conventional lifestyle interventions, such as calorie restriction and structured exercise, is particularly challenging within the confined and resource‐limited setting of a vessel (Hjarnoe and Leppin 2014; Mejsner et al. 2024). Therefore, there is a pressing need for effective, scalable nutritional strategies that can be seamlessly integrated into existing maritime food supply and dining routines.

Given this constraint, modifying the composition of commonly consumed foods presents a promising and feasible approach. Functional foods fortified with bioactive indigestible oligosaccharides are of particular interest, with Chitooligosaccharides, Mannose oligosaccharides and Fructo‐oligosaccharide being three subtypes widely studied for their metabolic regulatory properties (Yuan et al. 2024). These oligosaccharides resist upper gastrointestinal digestion and act as fermentable substrates for the gut microbiota, driving the production of short‐chain fatty acids and the modulation of systemic inflammation, which are key pathways underlying their well‐documented effects on host metabolic homeostasis (Ni et al. 2024; van Trijp et al. 2024; Zheng et al. 2025; Mutanda et al. 2014). Beyond this established prebiotic activity, preclinical and small‐scale human studies have linked these three oligosaccharide subtypes to the direct and indirect modulation of host lipid metabolism and energy balance (Mayang et al. 2025; Bessell et al. 2021; Zhang et al. 2022). However, evidence for their efficacy in improving body composition, particularly in reducing visceral adiposity as a key pathogenic fat depot, remains limited in high‐risk, non‐clinical occupational populations such as seafarers. While recent randomized controlled trials have demonstrated that fructo‐oligosaccharide supplementation can improve body composition and modulate gut microbiota in overweight and prediabetic populations, the effects on visceral fat specifically have not been well characterized (Le Bourgot et al. 2025). Furthermore, the systemic metabolic pathways that mediate the potential beneficial effects remain poorly characterized. Indeed, a recent trial by Li et al. (2025) reported that although fructo‐oligosaccharides reduced homocysteine levels, they failed to improve glycemic metrics, and the underlying metabolic pathways which include purine metabolism were only identified through functional prediction rather than direct mechanistic validation, leaving a significant gap in our mechanistic understanding of how these compounds act in metabolically challenged populations living in confined work environments.

To address this, we conducted a self‐controlled clinical trial in a cohort of male offshore seafarers with baseline metabolic risk. The primary objective was to evaluate the effects of a four‐week intervention with Chitooligosaccharides‐combination fortified noodles, a functional substitute for a dietary staple, on body composition with a focus on central adiposity. Secondarily, we employed untargeted metabolomics to map the associated systemic metabolic changes. We hypothesized that the intervention would lead to specific reductions in visceral fat and improvements in the lipid profile. Furthermore, we posited that these clinical benefits would be associated with and potentially mediated by distinct alterations in the serum metabolome, providing novel insights into the biological pathways engaged by dietary oligosaccharide supplementation in an at‐risk occupational population.

## 2 Materials and Methods

### 2.1 Ethics Statement

This study was conducted in accordance with the ethical principles set forth in the Declaration of Helsinki. The protocol was approved by the Ethics Review Committee of the Second Affiliated Hospital of Naval Medical University (Approval No.: 2024SL129, Shanghai, China) and registered with the Chinese Clinical Trial Registry on November 21, 2025 (Registration No.: ChiCTR2500112107). All participants provided written informed consent before participating in this study.

### 2.2 Study Design

**FIGURE 1:**
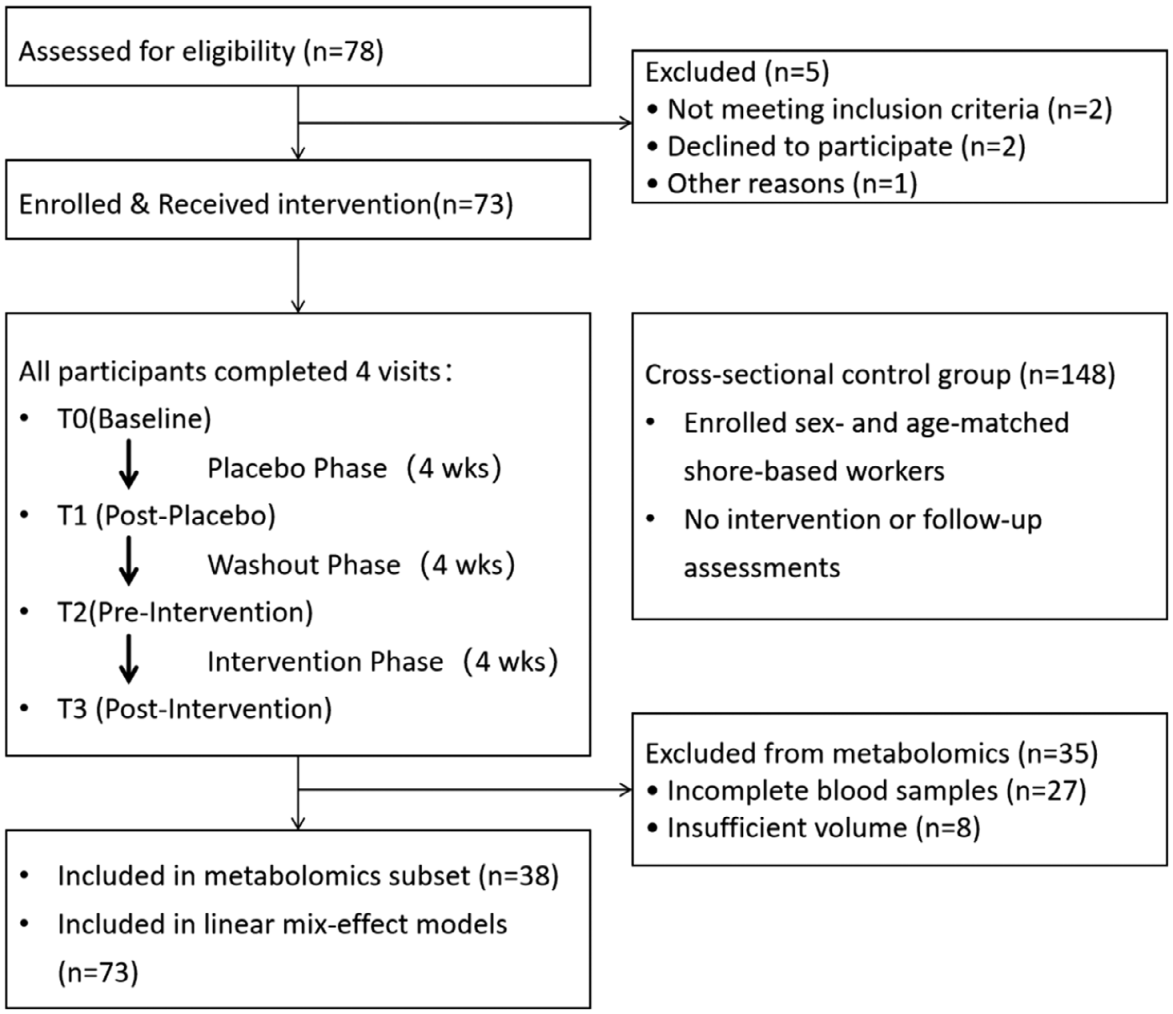
Flow diagram of participants through the study.

This self‐controlled study enrolled 78 healthy male seafarers aged 20–42 years. All participants were on consecutive offshore duty schedules, with a recent voyage duration exceeding 14 days and a shore break of no more than 3 days prior to enrollment. Participants were excluded if they met any of the following criteria: (1) had received any functional oligosaccharide supplementation (e.g., chitosan oligosaccharide, xylo oligosaccharides, fructo oligosaccharides) within the 6 months prior to enrollment; (2) had a known allergy to seafood, wheat gluten, or any other component of the study product; (3) had a habitual dietary fiber intake exceeding 30 g per day; or (4) followed a specific dietary pattern (e.g., vegan, ketogenic, low‐fiber, or thickened liquid diets). Prior to the intervention, five participants were excluded due to refusal of blood collection or other personal reasons. Consequently, 73 participants were included in the study. The study period consisted of a 4‐week placebo phase (Weeks 0–4), a 4‐week washout phase (Weeks 5–8), and a 4‐week intervention phase (Weeks 9–12). All participants completed the study, among whom 38 completed blood sample collection at all four time points. Additionally, 148 sex‐ and age‐matched shore‐based workers were enrolled as a cross‐sectional control (non‐interventional) group. The participant flow is detailed in Figure 1.

### 2.3 Placebo and Oligosaccharide Intervention

Both the placebo noodles and Chitooligosaccharides‐combination fortified noodles used in this study were provided by FuBanAn Food Co. Ltd. (Shanghai, China). The Chitooligosaccharides‐combination fortified noodles contained approximately 0.4 g Chitooligosaccharides, 0.2 g Mannose oligosaccharides, and 4 g Fructo‐oligosaccharide per 80 g serving, while their energy and other nutrient contents were comparable to those of the placebo noodles, as detailed in Table S1. Based on our preliminary research (Ji et al. 2021; Chen et al. 2022), participants were instructed to consume 80–100 g (raw weight) of the provided study noodles every other day during the placebo and intervention phases, replacing one staple meal, to minimize the impact of additional energy intake on the outcomes.

To account for individual variations in appetite, the researchers photographed participants' meal plates before and after each scheduled noodle serving to estimate and record the approximate intake. This evaluation confirmed that all participants consumed at least 80% of the noodles provided.

### 2.4 Dietary Intake and Physical Activity Assessment

During the study period, except for consuming the provided study noodles, participants received standardized meals from the ship galley and were instructed to maintain their habitual dietary patterns. Dietitians assessed their energy and nutrient intake using standardized 3‐day dietary records (including 2 week days and 1 weekend day). Changes in participants' physical activity were evaluated using the Global Physical Activity Questionnaire (GPAQ), and metabolic equivalent of task (MET) values were calculated accordingly. All the above data were collected at weeks 0, 4, 8, and 12 to allow for repeated‐measures assessment and to control for potential confounding effects of these lifestyle factors throughout the trial.

### 2.5 Clinical Measurements and Definition of Subgroups

All participants underwent body weight and waist circumference (WC) measurements at Weeks 0, 4, 8, and 12. Body composition, including body fat mass, visceral fat area (VFA), skeletal muscle mass (SMM), upper arm muscle circumference (AMC), and bone mineral content (BMC), was assessed using bioelectrical impedance analysis (BIA). The skeletal muscle mass index (SMI) was calculated as follows: SMI (kg/m^2^) = appendicular skeletal muscle mass (ASMM, kg)/height (m)^2^. Fasting blood samples were also collected to assess the lipid profile, including total cholesterol (TC), low‐density lipoprotein cholesterol (LDL‐C), high‐density lipoprotein cholesterol (HDL‐C), and triglycerides (TG).

Among the 38 participants who underwent metabolomic profiling (*n* = 38), based on the diagnostic criteria for central obesity, were stratified into subgroups using two established cut‐offs for males: (1) WC with an elevated threshold of > 90 cm (*n* = 10) versus normal WC (≤ 90 cm, *n* = 28); and (2) VFA with an elevated threshold of > 100 cm^2^ (*n* = 12) versus normal VFA (≤ 100 cm^2^, *n* = 26).

### 2.6 Enzyme‐Linked Immunosorbent Assay (ELISA)

Serum levels of leptin, adiponectin, fatty acid synthase (FAS), acetyl‐CoA carboxylase (ACC), peroxisome proliferator‐activated receptor alpha (PPAR‐α), and sterol regulatory element‐binding protein 1 (SREBP‐1) were measured using commercial ELISA kits purchased from MLBIO (Shanghai, China), according to the manufacturer's protocols.

### 2.7 Metabolomics Data Processing and Analysis

For serum samples, 200 μL was mixed with 600 μL of extraction solvent (methanol: acetonitrile = 1:1, v/v) and centrifuged at 12,000 *g* and 4°C for 10 min. The supernatant was evaporated to dryness, and the residue was reconstituted in a methanol–water solution (1:1, v/v). After centrifugation again under the same conditions, the supernatant was filtered through a membrane and subjected to liquid chromatography‐mass spectrometry (LC/MS) analysis. LC/MS analysis was performed as previously described (Liu et al. 2024), using a UPLC system (Shimadzu Inc., Kyoto, Japan) coupled to a Q Exactive Orbitrap mass spectrometer (Thermo Fisher Scientific Inc., MA, USA). Chromatographic separation was carried out on a UPLC HSS T3 column (2.5 μm, 100 mm × 2.1 mm, Waters, MA, USA) and a Waters UPLC BEH HILIC column (1.7 μm, 100 mm × 2.1 mm, Waters, MA, USA). The mobile phase consisted of 0.1% formic acid in water (phase A) and 0.1% formic acid in acetonitrile (phase B). The injection volume was set to 2 μL. QC samples and blank samples were inserted after each batch of samples to monitor system stability. The ion source was a heated electrospray ionization source, and full MS monitoring was performed over the mass range of *m*/*z* 100–1100.

Metabolomics data preprocessing and statistical analysis were performed using the MetaboAnalyst 6.0 web‐based platform (https://www.metaboanalyst.ca/) (Pang et al. 2024). The processed data were first normalized by median, log2‐transformed, and pareto‐scaled. Subsequently, multivariate analyses were conducted. Principal Component Analysis (PCA) was applied to the time‐series data under a one‐factor model to visualize general clustering trends. For supervised pattern recognition, Partial Least Squares‐Discriminant Analysis (PLS‐DA) was performed.

Primary screening for differential metabolites across time points was carried out using a time‐series linear mixed model (Murphy I et al. 2022; Artymowicz et al. 2023). The resulting *p*‐values were adjusted for multiple testing using the False Discovery Rate (FDR) correction. Metabolites with an FDR‐adjusted *p*‐value (*q*‐value) < 0.05 were considered statistically significant.

Functional interpretation of the significant metabolites was achieved through pathway analysis. Enrichment analysis and pathway topology analysis were conducted based on the Kyoto Encyclopedia of Genes and Genomes (KEGG) database. The significance of enriched pathways was also evaluated using FDR correction.

### 2.8 Statistical Analysis

We based our sample size calculation on previously published studies (Aoe et al. 2017; Baek I et al. 2023) and considered an anticipated change in visceral fat area after the intervention, with a power of 90% and a two‐sided alpha of 5%, as well as 20% attrition. This gave a sample size of 60 patients.

Data distribution was assessed for normality using the Shapiro–Wilk test. Continuous variables with normal distribution are presented as mean ± standard deviation, while non‐normally distributed data are expressed as median (interquartile range). Paired sample *t*‐tests were applied to evaluate within‐group changes over time (i.e., pre‐ vs. post‐intervention within each phase). A linear mixed model was fitted with time as a fixed effect and participant ID as a random effect to assess the net intervention effect. Statistical significance was defined as follows: *p < 0.05, **p < 0.01, and ***p < 0.001.

Furthermore, the Hidden Analysis of Latent Variable Associations (HALLA) (Ghazi et al. 2022) method was employed to identify associations between clinical outcomes and metabolomics data, specifically by correlating the net change in clinical parameters with the net change in metabolite levels, computed as (T3 − T2) − (T1 − T0). Additionally, Mantel tests were performed to evaluate the overall correlation between the net change of each individual metabolite and the combined net changes in all clinical indicators. All analyses were performed using R Studio (version 4.0; Posit Software, PBC) and SPSS (version 25.0; IBM Corp., Armonk, NY, USA).

## 3 Results

### 3.1 Baseline Characteristics of Study Participants

After excluding participants who did not meet the inclusion criteria, 73 participants were included in the analysis. The study cohort comprised entirely male offshore seafarers, with a median age of 24 (23, 26) years and a mean BMI of 24.28 ± 2.98 kg/m^2^. Baseline characteristics are summarized in Table 1. Compared to 148 age‐ and sex‐matched land‐based controls (Table S2), seafarers exhibited significantly higher body fat mass (17.99 ± 5.57 vs. 15.95 ± 5.69 kg, *p* < 0.05), body fat percentage (23.76 ± 4.93 vs. 20.86% ± 5.56%, *p* < 0.001), VFA (74.71 ± 25.08 vs. 66.68 ± 27.20 cm^2^, *p* < 0.05), and LDL‐C (2.71 ± 0.69 vs. 2.43 ± 0.61 mmol/L, *p* < 0.005). In contrast, AMC (26.53 ± 1.87 vs. 27.03 ± 1.63 cm, *p* < 0.05), SMM (31.59 ± 4.14 vs. 33.36 ± 3.71 kg, *p* < 0.005), and BMC (3.10 ± 0.43 vs. 3.28 ± 0.41 kg, *p* < 0.005) were significantly lower. This pattern of elevated adiposity coupled with reduced lean mass underscores the unique health challenges faced by offshore seafarers, pointing to an elevated risk of central obesity and related metabolic disorders in this occupational group.

**TABLE 1:** Baseline characteristics of the study participants.

Characteristic	All Participants (*N* = 73)
Demographics	
Age (years)	24.00 (23.00, 26.00)
Sex, *n* (%)	74 (100) Male
Length of service (years)	5.55 ± 4.74
Anthropometrics	
Height (m)	1.75 ± 0.05
Body weight (kg)	74.42 ± 10.87
Body mass index (kg/m²)	24.28 ± 2.98
Waist circumference, WC (cm)	80.79 ± 7.60
Body composition (by BIA)	
Body fat mass (kg)	17.99 ± 5.57
Body fat percentage (%)	23.76 ± 4.93
Visceral fat area, VFA (cm²)	74.71 ± 25.08
Skeletal muscle mass, SMM (kg)	31.59 ± 4.14
Skeletal muscle index,SMI (kg/m²)	8.18 ± 0.76
Upper arm muscle circumference, AMC (cm)	26.53 ± 1.87
Bone mineral content (kg)	3.10 ± 0.43
Serum biochemical parameters	
Leptin (pg/ml)	99.9 ± 51.8
Adiponectin (pg/ml)	35.7 ± 13.5
Serum total cholesterol, TC (mmol/L)	4.47 ± 0.83
Serum Triglyceride, TG (mmol/L)	1.11 ± 0.61
Serum low‐density lipoprotein cholesterol, LDL‐C (mmol/L)	2.71 ± 0.69
Serum high‐density lipoprotein cholesterol, HDL‐C (mmol/L)	1.30 ± 0.34
Serum albumin (g/L)	47.49 ± 1.92
Diastolic blood pressure (mmHg)	74.34 ± 8.45
Systolic blood pressure (mmHg)	117.37 ± 10.18
Glycated hemoglobin, HbA1c (%)	5.41 ± 0.31
High‐sensitivity C‐reactive protein, hs‐CRP (mg/L)	0.50(0.25,0.80)
Serum creatinine (μmol/L)	82.40 ± 7.74
Urea (mmol/L)	5.87 ± 1.21
Dietary intake (24‐hour recall)	
Energy intake (kcal/day)	3075.67 ± 973.27
Fiber intake (g/day)	21.37 ± 16.37
Physical Activity	
Total physical activity (MET‐min/week)	2279.00 (693.00, 3980.00)

### 3.2 Chitooligosaccharides‐Combination Fortified Noodles Ameliorated Central Obesity and Improved Body Composition Compared to Placebo

To evaluate the specific effects of the oligosaccharide intervention, we compared the changes (Δ) in primary clinical outcomes between the 4‐week placebo phase (T0 to T1) and the 4‐week intervention phase (T2 to T3). The efficacy of the washout period was confirmed, as no significant carryover effects were observed for any main parameters at T2 (*p* > 0.05, Figure S1 for individual trajectories of all clinical outcomes across the four time points). Furthermore, both dietary energy intake and physical activity levels, assessed as potential confounders, showed no significant differences across the four assessment time points (*p* > 0.05, Figure S1).

**FIGURE 2:**
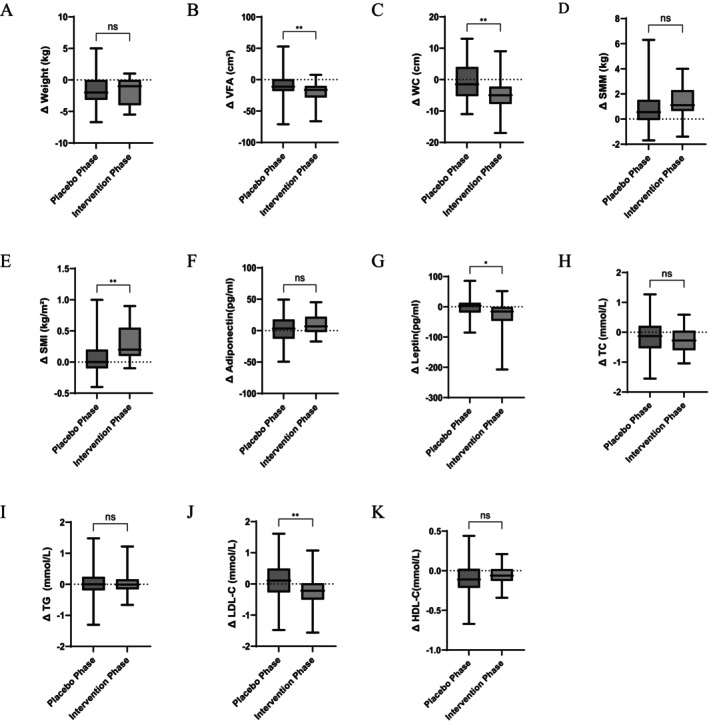
Chitooligosaccharides‐combination fortified noodles ameliorated central obesity and improved body composition compared to placebo. (A–K) Box plots comparing the magnitude of change (Δ) in clinical outcomes between the 4‐week placebo phase (T0 to T1) and the 4‐week intervention phase (T2 to T3). Δ values were calculated as the difference between the end and start of each respective phase. *p* values indicate comparisons of the Δ values between the two phases using paired *t*‐tests or Wilcoxon signed‐rank tests as appropriate. VFA, visceral fat area; WC, waist circumference; SMM, skeletal muscle mass; SMI, skeletal muscle mass index; TC, total cholesterol; TG, triglycerides; LDL‐C, low‐density lipoprotein cholesterol; HDL‐C, high‐density lipoprotein cholesterol. *p < 0.05, **p < 0.01.

Compared with the placebo phase, consumption of Chitooligosaccharides‐combination fortified noodles for 4 weeks showed no significant change in body weight (ΔWeight: −1.70 ± 1.88 vs. −1.47 ± 2.56 kg, *p* > 0.05, Figure 2A). However, there were significant improvements in visceral fat area (ΔVFA: −19.25 ± 15.06 vs. −7.47 ± 18.73 cm^2^, *p* < 0.005, Figure 2B), waist circumference (ΔWC: −5.10 ± 5.32 vs. −1.04 ± 5.98 cm, *p* < 0.005, Figure 2C) and skeletal muscle mass index (ΔSMI: 0.29 ± 0.29 vs. 0.08 ± 0.25 kg/m^2^, *p* < 0.005, Figure 2E). Furthermore, the intervention also yielded beneficial metabolic effects, including reduced serum levels of leptin (ΔLeptin: −25.26 ± 51.40 vs. −5.04 ± 31.57 pg/mL, *p* < 0.05, Figure 2G) and low‐density lipoprotein cholesterol (ΔLDL‐C: −0.19 ± 0.48 vs. 0.13 ± 0.58 mmol/L, *p* < 0.005, Figure 2J). However, no significant changes were observed in the serum levels of FAS, ACC, PPAR‐α, or SREBP‐1 following the intervention (*p* > 0.05), as shown in Figure S2.

### 3.3 Linear Mixed Model Confirms the Beneficial Effects on Central Adiposity

A linear mixed model was employed to quantify the net intervention effect, accounting for intra‐individual correlations across time points. As shown in Table 2, a significant net reduction was observed in VFA (*β* = −18.36, 95% CI: −31.56 to −5.16; *p* < 0.05), WC (*β* = −7.51, 95% CI: −10.78 to −4.23; *p* < 0.001) and LDL‐C (*β* = −0.33, 95% CI: −0.55 to −0.11; *p* < 0.005). While the body weight, total body fat mass, skeletal muscle mass, adipokine levels (leptin and adiponectin), or the lipid profile (including TC, TG, and HDL‐C) showed no statistically significant improvements in the mixed models.

**TABLE 2:** Effects of the Intervention on Outcome Measures from Linear Mixed Models (Interaction Term).

Outcome measure	Net effect (β)	SE	95% CI	*t* value	*p* value
Weight (kg)	−3.57	2.92	−9.32, 2.19	−1.22	0.223
**VFA (cm^2^**)	−**18.36**	**6.76**	−**31.69, −5.03**	−**2.72**	**< 0.05**
**WC (cm)**	−**7.51**	**1.68**	−**10.80,−4.21**	−**4.48**	**< 0.001**
Body fat mass (kg)	−1.67	1.43	−4.49, 1.15	−1.17	0.244
SMM (kg)	0.14	1.20	−2.22, 2.50	0.12	0.905
SMI (kg/m^2^)	0.16	0.22	−0.28, 0.59	0.71	0.479
Leptin (pg/ml)	−12.80	14.83	−42.11, 16.50	−0.86	0.389
Adiponectin (pg/mL)	6.80	4.60	−2.29, 15.90	1.48	0.142
TC (mmol/L)	−0.24	0.23	−0.69, 0.21	−1.06	0.290
TG (mmol/L)	−0.29	0.16	−0.61, 0.03	−1.79	0.076
**LDL‐C (mmol/L)**	−**0.33**	**0.11**	−**0.55**, −**0.11**	−**3.03**	**< 0.005**
HDL‐C (mmol/L)	0.10	0.08	−0.06, 0.25	1.22	0.225

*Note:* Bold values indicate statistically significant differences in the intervention effects between groups (*p* < 0.05).

### 3.4 Serum Metabolomic Profiling and Screening of Differential Metabolites

**FIGURE 3:**
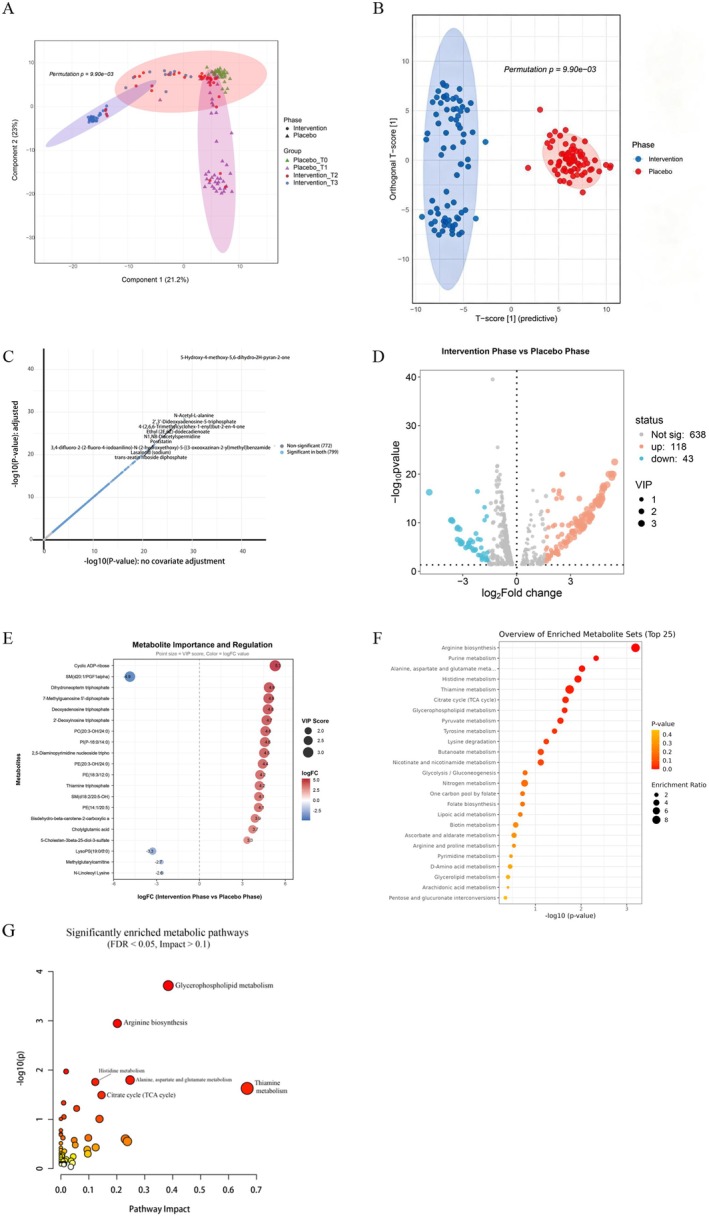
Serum metabolomic profiling and screening of differential metabolites. (A) PLS‐DA score plot based on the time‐series + one‐factor model. (B) OPLS‐DA score plot for the discrimination of the placebo phase and intervention phase. (C) Primary screening of differential metabolites via time‐series linear mixed model. (D) Volcano plot of differential metabolites between oligosaccharide intervention and placebo groups. (E) VIP ranking of top 15 metabolites. (F, G) KEGG pathway enrichment and analysis of core differential metabolites.

Untargeted serum metabolomics was performed on a subset of 38 participants who completed serum sample collection at all four time points (T0–T3). Their baseline characteristics did not differ significantly from those of the excluded participants (Table S3). Partial least squares‐discriminant analysis (PLS‐DA) incorporating treatment group and time point revealed a clear separation between the placebo and oligosaccharide intervention phases, indicating a global shift in the metabolic profile (Figure 3A). This metabolic separation was further verified using orthogonal partial least squares discriminant analysis (OPLS‐DA) (Figure 3B). The robustness of this separation was independently confirmed by OPLS‐DA score plots comparing the placebo phase (T1) versus the intervention phase (T3) and the pre‐intervention time point (T2) versus post‐intervention (T3), as shown in Figure S3A,B, respectively.

Identification of intervention‐altered metabolites relied on a time‐series linear mixed model (Murphy I et al. 2022; Artymowicz et al. 2023), which initially yielded 799 candidate features (Figure 3C). Applying thresholds (|log_2_FC| > 1, VIP > 1, FDR < 0.05) refined these to 161 differential metabolites (118 upregulated, 43 downregulated) between the two treatment groups (Figure 3D). To pinpoint key drivers of this separation, metabolites were ranked by VIP scores. The top 20 VIP‐ranked metabolites are displayed in Figure 3E. Strikingly, 16 of these top contributors were upregulated during the intervention. Cyclic ADP‐ribose (VIP = 3.32, log_2_FC = 5.3) and the sphingolipid SM(d20:1/PGF1α) (VIP = 3.07, log_2_FC = −4.9) were identified as the two most influential discriminators.

To delineate the functional associations of these key metabolites, we performed metabolite set enrichment analysis. The results revealed a broad rewiring of central metabolism. Pathways related to amino acid metabolism (e.g., Arginine and Proline metabolism, Histidine metabolism), nucleotide metabolism (Purine and Pyrimidine metabolism), and energy metabolism (Citrate cycle, Glycolysis, and Thiamine metabolism) were significantly enriched (Figure 3F).

Subsequently, KEGG pathway topology analysis was employed to pinpoint the most impactful pathways. This analysis integrated both the statistical significance (*p*‐value) and the pathway impact score derived from betweenness centrality. As shown in Figure 3G, Glycerophospholipid metabolism emerged as the most significantly altered pathway, exhibiting the highest pathway impact, followed by Arginine biosynthesis and Thiamine metabolism. The convergence of these three independent analyses—VIP ranking, enrichment, and topology impact—robustly highlights glycerophospholipid, amino acid metabolism, and thiamine metabolism as central to the oligosaccharide intervention's metabolic effects.

### 3.5 Integration of Metabolomic and Clinical Phenotypes Reveals Key Metabolic‐Clinical Associations

**FIGURE 4:**
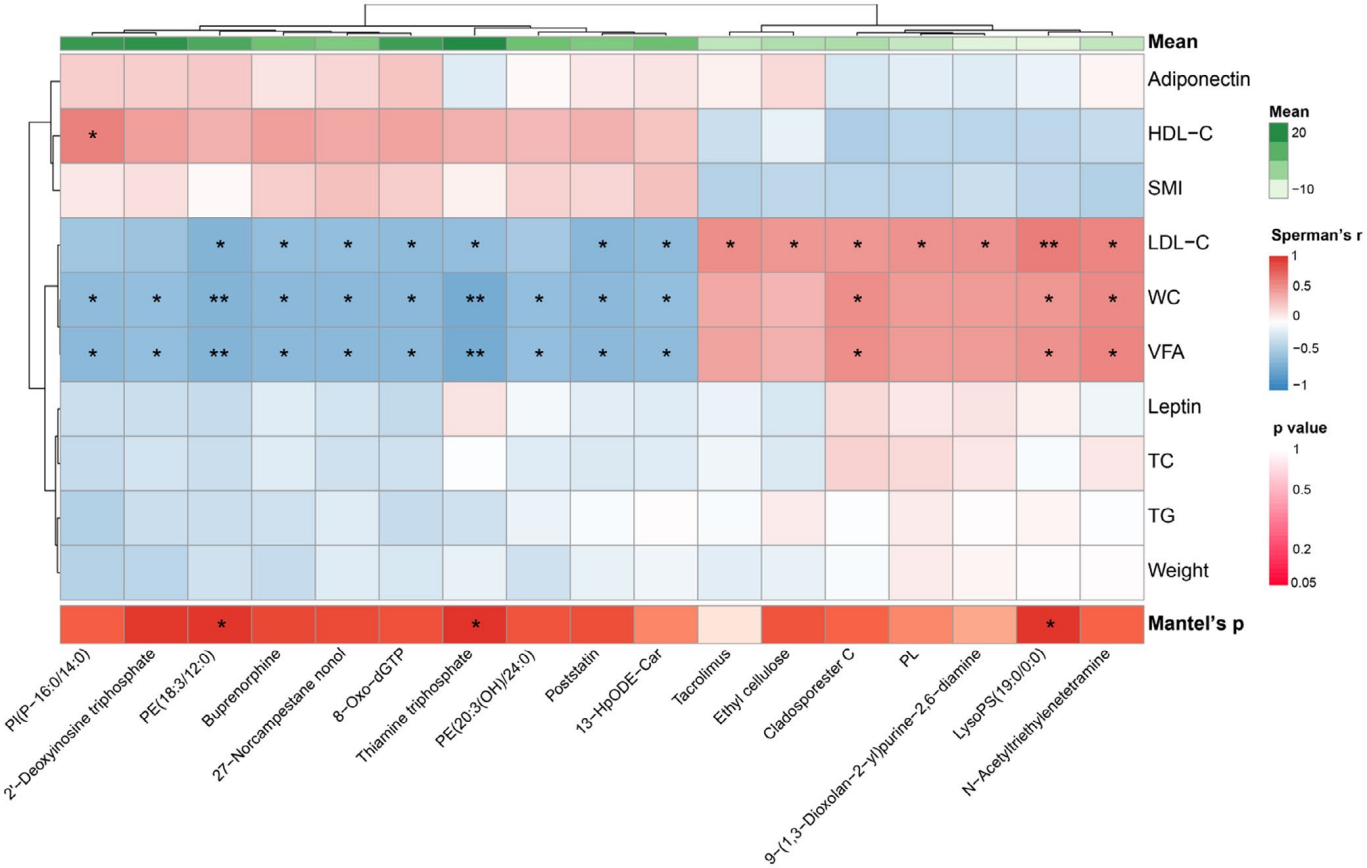
Integrated associations between serum metabolites and clinical phenotypes. The heatmap displays pairwise correlations between intervention‐induced net changes in serum metabolites and clinical parameters, analyzed using the Hidden Analysis of Latent Variable Associations (HALLA). The color gradient represents the Spearman correlation coefficient (red: Positive correlation; blue: Negative correlation). The top row shows the net change in mean metabolite levels (Mean). The bottom row presents Mantel's *p*‐values, assessing the overall correlation between each metabolite and the coordinated pattern of changes across all clinical indicators. *FDR‐adjusted *p* < 0.05, **FDR‐adjusted *p* < 0.005.

The HALLA analysis revealed multiple significant metabolite–clinical associations (*FDR* < 0.05), with distinct correlation patterns between metabolites and clinical variables (Figure 4). Among these, the most prominent negative correlations were observed between metabolites from the core perturbed pathways—glycerophospholipid and thiamine metabolism—and improvements in central adiposity and lipid profiles. Specifically, the net changes in thiamine triphosphate (ThTP), the biologically active phosphorylated form of thiamine (KEGG ID: C03028, Figure S4A depicts its position within the enriched thiamine metabolism pathway network), and phosphatidylethanolamine PE(18:3/12:0), a key structural glycerophospholipid involved in membrane homeostasis and mitochondrial energy metabolism (KEGG ID: C00350, Figure S4B depicts its position within the enriched Glycerophospholipid metabolism pathway network), were significantly inversely correlated with the net changes in VFA, WC, and LDL‐C, indicating that intervention‐induced upregulation of these metabolites was accompanied by improvements in adiposity and atherogenic lipid profiles. A secondary negative association was also observed for two additional functional glycerophospholipids, PE(20:4‐OH(5S)/16:0) and PI(P‐16:0/14:0), whose net changes were specifically negatively correlated with the net changes in VFA and WC. In contrast, the strongest positive correlation was observed for LysoPS(19:0/0:0), a lysophospholipid metabolite from the degradation branch of glycerophospholipid metabolism. The net change of this metabolite after intervention showed a robust positive correlation with the net changes in VFA, WC, and LDL‐C, meaning that the intervention‐induced reduction of this pro‐adipogenic lipid mediator was synchronized with the decreases in central adiposity and atherogenic lipid levels.

Complementing these pairwise associations, Mantel tests were performed to assess how each metabolite correlated with the coordinated changes across the full set of clinical indicators (Figure 4). While several metabolites showed significant individual correlations with clinical parameters in HALLA, only PE(18:3/12:0), thiamine triphosphate, and LysoPS(19:0/0:0) exhibited a significant overall association with the global pattern of clinical improvement. These results indicates these three metabolites are not only correlated with individual clinical outcomes but also reflect the broader, systemic metabolic rewiring that underlies the intervention‐related clinical benefits.

### 3.6 Differential Responses of Key Metabolites in Subgroups With Normal Versus Elevated Visceral Adiposity

To assess differential metabolic responses to the intervention across adiposity status, we compared changes in the three key metabolites identified in the preceding multi‐omics association analyses between participants with normal versus elevated visceral fat area (VFA) or waist circumference (WC).

**FIGURE 5:**
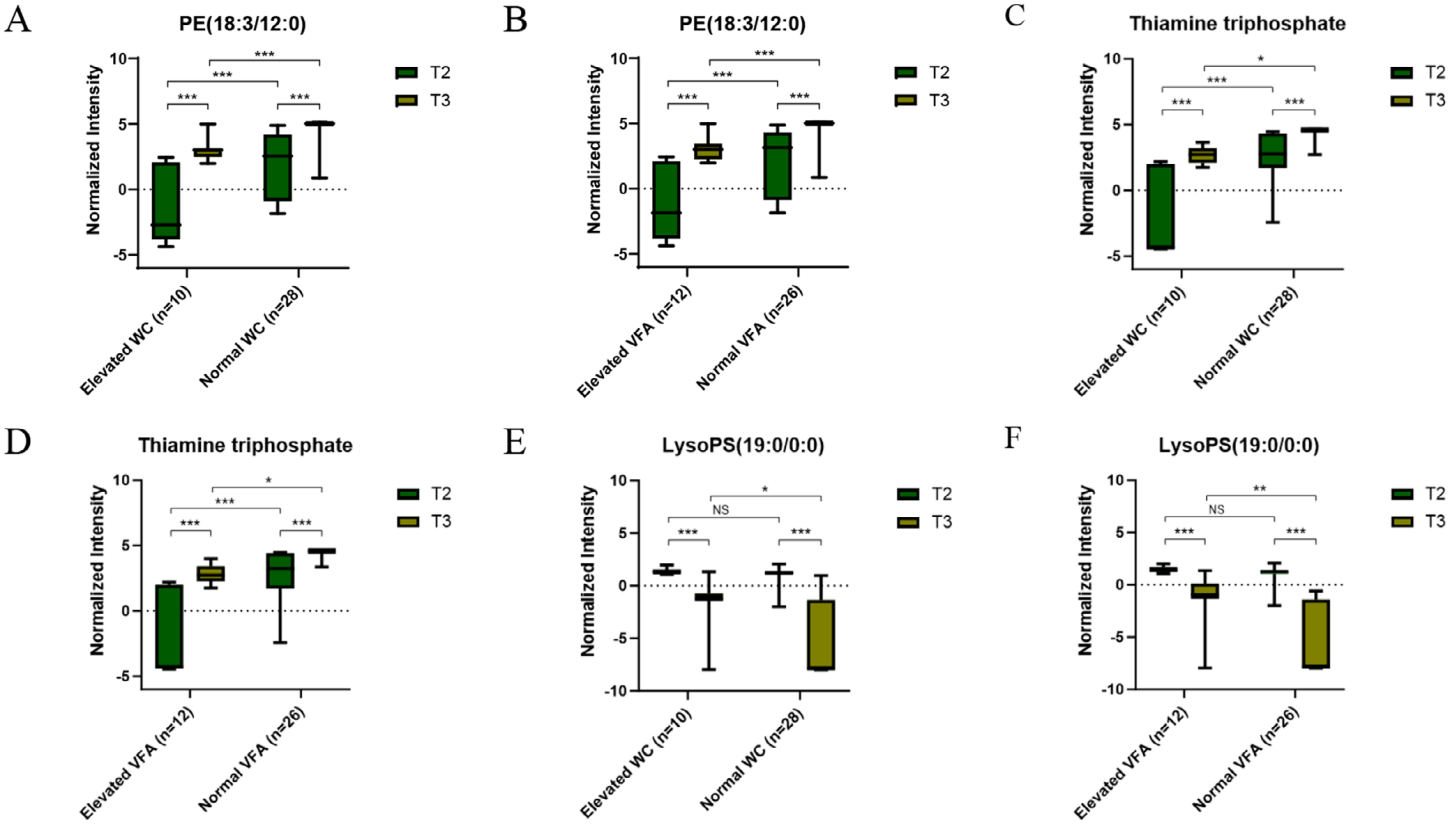
Differential responses of key metabolites in subgroups with normal versus elevated visceral adiposity. (A–F) Box plots depicting normalized metabolite intensities at T2 (pre‐intervention, after the 4‐week washout) and T3 (post‐intervention) in participants stratified by waist circumference (WC; elevated > 90 cm, *n* = 10; normal ≤ 90 cm, *n* = 28) and visceral fat area (VFA; elevated > 100 cm^2^, *n* = 12; normal ≤ 100 cm^2^, *n* = 26). Asterisks above brackets denote within‐group comparisons between T2 and T3 (paired *t*‐test); NS or asterisks above horizontal bars connecting the two subgroups denote between‐group comparisons at the indicated time point (independent *t*‐test or Mann–Whitney *U* test). PE, phosphatidylethanolamine; ThTP, thiamine triphosphate; LysoPS, lysophosphatidylserine. *p < 0.05, **p < 0.005, ***p < 0.001.

As shown in Figure 5, the levels of PE(18:3/12:0) and ThTP were significantly lower in participants with elevated adiposity compared to those with normal adiposity at T2 (*p* < 0.001, Figure 5A–D). The oligosaccharide intervention significantly increased their levels within this subgroup (*p* < 0.001). Notably, the magnitude of increase (Δ) for both metabolites was greater in the elevated adiposity subgroup than in the normal subgroup (*p* < 0.05, data not shown in figure), indicating a more pronounced corrective response. Despite this greater change, their absolute levels at T3 remained lower than in the normal subgroup (*p* < 0.05), reflecting the persistence of the baseline deficit. In contrast, no significant baseline difference was observed for lysophosphatidylserine (LysoPS) (19:0/0:0) (*p* > 0.05, Figure 5E,F). Following the intervention, LysoPS(19:0/0:0) levels were significantly reduced in the elevated adiposity subgroup (*p* < 0.05).

## 4 Discussion

This self‐controlled trial demonstrated that a 4‐week dietary intervention with Chitooligosaccharides‐combination fortified noodles significantly improved body composition in a cohort of male offshore seafarers, a population characterized by an unfavorable metabolic profile at baseline (Baygi et al. 2016; Jiang et al. 2022; Jepsen and Rasmussen 2016). The intervention led to marked reductions in central adiposity, as evidenced by decreases in VFA and WC, alongside favorable improvements in leptin, LDL‐C, and SMI. These clinical improvements were underpinned by a systemic rewiring of the serum metabolome, most notably in glycerophospholipid and thiamine metabolism pathways. The integration of metabolomic and clinical data further revealed that changes in specific metabolites from these pathways were strongly correlated with improvements in adiposity and atherogenic lipid profiles. Collectively, these findings provide preliminary evidence supporting the potential of targeted oligosaccharide supplementation as a practical nutritional strategy to mitigate central obesity and its associated metabolic risks in this high‐risk occupational group.

The observed improvements in central obesity without weight loss are clinically meaningful. Visceral adipose tissue, rather than total adiposity, is a key driver of insulin resistance and cardiometabolic risk (Antonio‐Villa et al. 2023; Bensussen et al. 2023; Neeland I et al. 2019). In this study, the estimated net reduction in VFA (approximately 18 cm^2^) after four weeks is comparable to effects reported with structured lifestyle interventions and is likely sufficient to confer metabolic benefits, particularly given the concurrent significant reduction in LDL‐C (He et al. 2022; Danielsen et al. 2013). Indeed, these findings align with the accumulating body of evidence on the metabolic effects of functional oligosaccharides and prebiotics. Previous studies have shown that certain compounds, such as inulin‐type fructans and fructo‐oligosaccharides, can modestly improve abdominal adiposity, body fat percentage, and waist circumference (Ziaei et al. 2024; Nicolucci et al. 2017), as well as lipid profiles, including reductions in LDL‐cholesterol and triglycerides (Costa et al. 2015). For seafarers, whose occupational environment imposes high caloric demands alongside limited access to fresh, nutrient‐dense foods (Zyriax et al. 2018; Neumann et al. 2021), an intervention that simply replaces a staple food (noodles) with a functional alternative offers a feasible and scalable approach to metabolic health.

Mechanistically, chitooligosaccharides exert typical prebiotic effects by escaping upper intestinal digestion and reaching the colon to modulate gut microbial composition. They enrich beneficial commensal bacteria and boost microbial SCFA production, which facilitates intestinal GLP‐1 secretion, modulates key hepatic lipid‐metabolizing enzymes, and maintains steady hepatic lipid metabolism (Wang et al. 2020). Beyond gut microbiota‐dependent regulation, composite oligosaccharides also exert direct metabolic benefits. They can inhibit peripheral lipogenesis, enhance fatty acid oxidation, and alleviate chronic low‐grade inflammation in adipose tissue, a key driver of central adiposity and dyslipidemia (Zhou et al. 2023). Combined with these gut‐dependent and independent pathways, the long‐term intake of chitooligosaccharides‐combination fortified noodles effectively attenuates visceral fat accumulation and improves circulating lipid profiles.

The metabolomic data provide insight into the pathways underlying these clinical improvements. Glycerophospholipid metabolism was the most significantly altered pathway, aligning with considerable evidence that connects its dysregulation to obesity and insulin resistance. The alterations in glycerophospholipid metabolism could be attributed to gut microbiota‐mediated mechanisms, given that functional oligosaccharides are known to modulate host lipid metabolism via microbial pathways (Wu et al. 2025). Glycerophospholipids are fundamental components of cellular membranes and play crucial roles in signal transduction and energy homeostasis. The oligosaccharides intervention‐induced upregulation of phosphatidylethanolamine species, particularly PE(18:3/12:0) and the downregulation of the lysophospholipid LysoPS(19:0/0:0) suggest a shift in membrane lipid composition and signaling. Phosphatidylethanolamines rich in polyunsaturated fatty acids are known to support mitochondrial membrane integrity and function. Given that mitochondrial biogenesis and uncoupled respiration are central to brown adipose tissue thermogenesis and energy expenditure (Johnson et al. 2023; Su et al. 2023; Cheng et al. 2021), the increase in PE(18:3/12:0) could potentially indicate an enhanced capacity for lipid oxidation and energy dissipation, thereby contributing to the reduction in central adiposity. Furthermore, KEGG pathway analysis revealed that the oligosaccharide intervention significantly impacted multiple nodes within the glycerophospholipid metabolism network. The differentially abundant metabolites were distributed across key sub‐pathways, including those for phosphatidylcholine, phosphatidylethanolamine, and lysophospholipids. This coordinated remodeling highlights a broad effect on lipid metabolism that extends beyond simple storage or mobilization, implicating systemic roles in membrane biology, signal transduction, and cellular energy sensing.

Concurrently, we observed a significant enrichment in thiamine (vitamin B1) metabolism, with ThTP showing a robust increase after oligosaccharide consumption. This activation is physiologically significant, as thiamine serves as an indispensable cofactor for mitochondrial energy production, fundamentally driving ATP generation and supporting cellular metabolic homeostasis. Moreover, emerging evidence suggests extracellular thiamine enhances the thermogenic capacity of human adipocytes, increasing mitochondrial respiration and the expression of key genes like UCP1 (Vinnai et al. 2023). Thus, sustaining adequate thiamine status is essential for supporting these metabolic processes. However, the seafaring population studied here faces a heightened risk of thiamine deficiency due to a voyage diet that relies heavily on polished rice and refined grains, which are poor sources of the vitamin (Schostak et al. 2023; Doung‐ngern et al. 2007). In this context, the oligosaccharide intervention may have augmented thiamine availability through two complementary mechanisms. First, oligosaccharides can serve as fermentable substrates to support colonic microbial thiamine synthesis, as demonstrated in animal models (He et al. 2026; Wan et al. 2022). The second mechanism remains hypothetical: the increase in ThTP, itself a mitochondrial metabolite, could enhance mitochondrial bioenergetics, or vice versa. Thus, a reciprocal relationship between thiamine metabolism and mitochondrial function may exist, but this requires future validation.

HALLA analysis established precise quantitative correlations between intervention‐triggered metabolic variations and individual clinical phenotypic improvements, while complementary Mantel tests further validated the global association between key differential metabolites and coordinated changes in the overall clinical indicator spectrum. The strong negative correlations between the increases in PE(18:3/12:0) and thiamine triphosphate with decreases in VFA, WC, and LDL‐C are particularly noteworthy. This suggests that individuals who experienced a greater upregulation of these potentially beneficial metabolites also achieved greater reductions in central adiposity and atherogenic lipids. The association of elevated PE(18:3/12:0) with reduced adiposity aligns with its putative role in supporting mitochondrial function and lipid oxidation (Johnson et al. 2023; Gangolf et al. 2010; Bettendorff 2021), while the rise in ThTP corroborates improved thiamine‐dependent energy metabolism. Conversely, the reduction in LysoPS(19:0/0:0) was positively correlated with clinical improvements. These robust metabolite‐clinical associations strengthen the biological plausibility that the observed metabolic rewiring is not merely an epiphenomenon but is functionally involved in driving the clinical benefits. This “molecular‐phenotype bridge” offers a plausible explanatory framework for how a dietary oligosaccharide intervention can translate into measurable health outcomes.

The stratified analysis based on baseline central obesity status revealed a critical nuance: participants with elevated VFA or WC at baseline exhibited more pronounced dysregulation in the key metabolites (lower PE(18:3/12:0) and thiamine triphosphate, higher LysoPS) and demonstrated a more significant correction following the intervention. This pattern indicates that the oligosaccharide intervention acted primarily by correcting a pre‐existing metabolic imbalance rather than merely providing a uniform “boost.”“Individuals with central obesity, who presumably had a greater degree of metabolic dysfunction, derived a more substantial corrective benefit. This finding has important implications for precision nutrition. It suggests that oligosaccharide‐based interventions may be particularly efficacious for individuals or occupational groups, like seafarers, who exhibit a phenotype of central obesity and related metabolic disturbances, allowing for targeted, risk‐stratified dietary recommendations. A key strength of this study is its use of a self‐controlled design with a washout period, which allowed each participant to serve as their own control, thereby reducing between‐subject variability. The use of linear mixed models further strengthened the analysis by accounting for repeated measures and testing for treatment–time interactions. In addition, the integration of untargeted metabolomics with clinical phenotyping enabled a data‐driven approach to identify specific metabolites that link the intervention to metabolic outcomes, rather than relying on a priori hypotheses.

This study has several limitations. First, the modest sample size, particularly for the metabolomics subset, may have limited statistical power for some analyses; moreover, subgroup analyses by baseline central adiposity were exploratory and should be regarded as hypothesis‐generating. Second, the self‐controlled crossover design reduces inter‐individual variability but cannot fully exclude temporal or period effects, and the four‐week intervention captures early metabolic adaptation only, precluding conclusions on long‐term sustainability, body‐weight changes, or generalizability to female seafarers, other age groups, and land‐based populations. Third, bioelectrical impedance analysis estimates visceral fat area with lower precision than computed tomography or magnetic resonance imaging, so VFA changes should be interpreted as approximate clinical signals. Fourth, the metabolite–outcome associations identified do not establish causality, and direct experimental evidence is needed to determine whether changes in ThTP, PE(18:3/12:0), or LysoPS(19:0/0:0) are mediators or merely markers of metabolic improvement. Finally, the role of the gut microbiota was not assessed; given that oligosaccharides are known microbiota modulators, future metagenomic studies are essential to elucidate whether the observed metabolic remodeling is microbially mediated.

## 5 Conclusions

This study demonstrates that a four‐week dietary intervention with Chitooligosaccharides‐combination fortified noodles effectively reduced visceral fat accumulation and waist circumference, improved skeletal muscle mass index, and lowered circulating LDL‐C and leptin levels without significant alterations in overall body weight among young male offshore seafarers. These clinical benefits were associated with targeted remodeling of glycerophospholipid and thiamine metabolism, with PE(18:3/12:0), thiamine triphosphate, and LysoPS(19:0/0:0) identified as core regulatory metabolites. Participants with elevated baseline visceral adiposity exhibited greater metabolic responsiveness, underscoring the potential of this food‐based strategy for precision nutrition in high‐risk occupational cohorts. These findings provide preliminary evidence for a practical nutritional intervention to mitigate cardiometabolic risk in seafarers, although future randomized controlled trials are warranted to validate long‐term efficacy and elucidate the role of gut microbiota.

## Author Contributions

**Jiaming Wang:** data curation, investigation. **Zhongbo Bian:** writing – original draft, visualization, data curation, investigation. **Xinjie Zhou:** investigation. **Yi Lu:** software, data curation. **YanYing Yao:** investigation, visualization. **Juan Li:** writing – review and editing, funding acquisition, resources, project administration. **Xiao guo Ji:** project administration, investigation, supervision. **Xiangru Feng:** methodology, supervision.

## Funding

This study was supported by scientific research project of the Shanghai Municipal Science and Technology Commission (24Y12801100).

## Conflicts of Interest

The authors declare no conflicts of interest.

## Supporting information


**Figure S1:** Chitooligosaccharides‐combination fortified noodles ameliorated central obesity and improved body composition compared to placebo.
**Figure S2:** Chitooligosaccharides‐combination fortified noodles ameliorated central obesity and improved body composition compared to placebo.
**Figure S3:** OPLS‐DA score plots of serum metabolomics.
**Figure S4:** Integration of Metabolomic and Clinical Phenotypes Reveals Key Metabolic‐Clinical Associations.
**Table S1:** Nutritional composition of placebo and Chitooligosaccharides‐combination fortified noodles.
**Table S2:** Baseline characteristics of land‐based controls and offshore seafarers.
**Table S3:** Baseline Characteristics of Participants Included versus Excluded from Metabolomics Analysis.

## Data Availability

The data that support the findings of this study are available on request from the corresponding author. The data are not publicly available due to privacy or ethical restrictions.
