# Supplementary material for: Effect of Chitooligosaccharides‐Combination Fortified Noodles Intervention on Visceral Adiposity and Serum Metabolites in a High‐Risk Occupational Cohort of Young Seafarers
Source: Food Sci Nutr. 2026 Jul 25;14(7):e72101. doi: 10.1002/fsn3.72101 (PMC13401165; doi:10.1002/fsn3.72101)
Supplement: Supplementary file 1 — Figure S1: Chitooligosaccharides‐combination fortified noodles ameliorated central obesity and improved body composition compared to placebo. Figure S2: Chitooligosaccharides‐combination fortified noodles ameliorated central obesity and improved body composition compared to placebo. Figure S3: OPLS‐DA score plots of serum metabolomics. Figure S4: Integration of Metabolomic and Clinical Phenotypes Reveals Key Metabolic‐Clinical Associations. Table S1: Nutritional composition of placebo and Chitooligosaccharides‐combination fortified noodles. Table S2: Baseline characteristics of land‐based controls and offshore seafarers. Table S3: Baseline Characteristics of Participants Included versus Excluded from Metabolomics Analysis. [file FSN3-14-e72101-s001.docx]

| A | 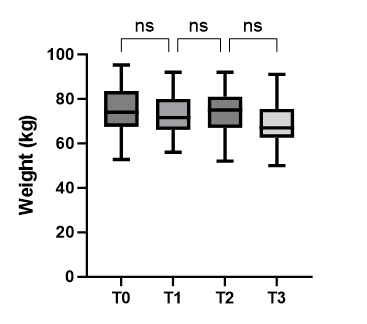 | B | 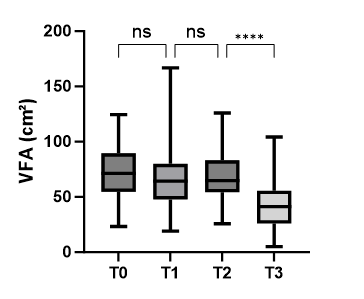 | C | 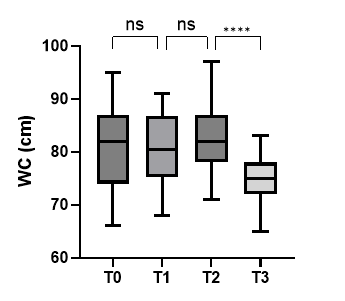 |
| --- | --- | --- | --- | --- | --- |
| D | 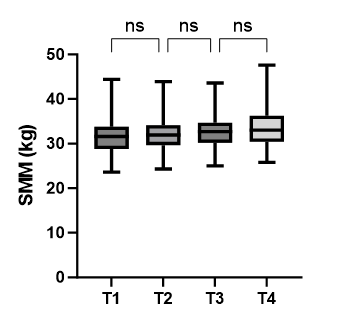 | E | 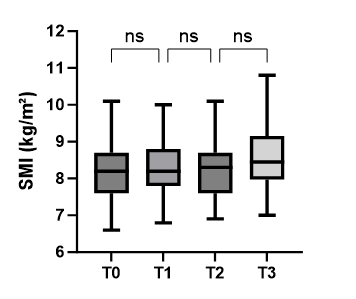 | F | 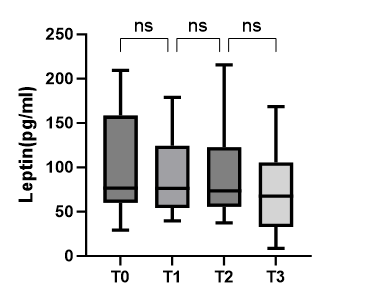 |
| G | 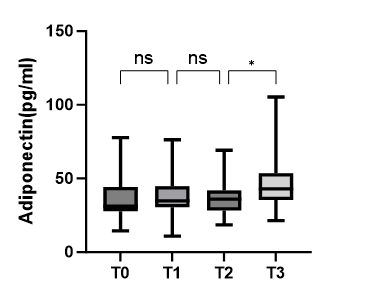 | H | 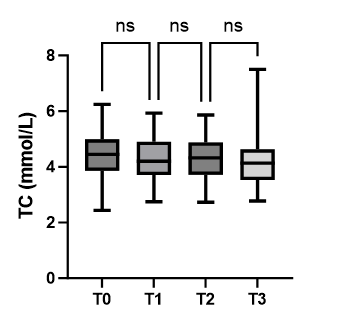 | I | 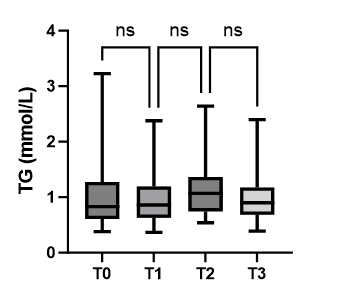 |
| J | 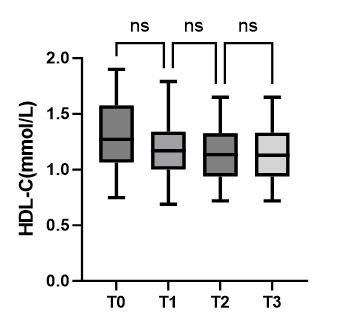 | K | 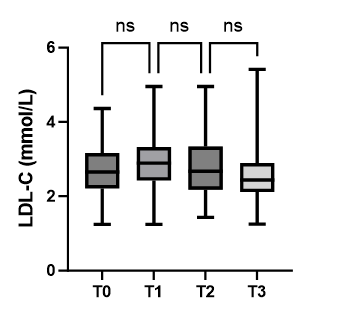 | L | 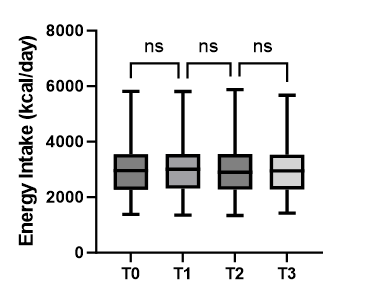 |
| M | 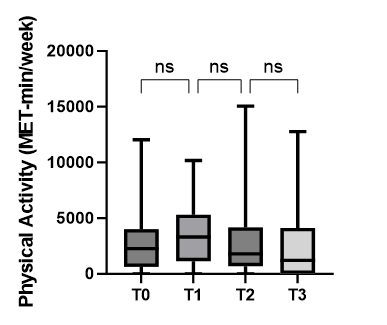 |  |  |  |  |

**Figure S1. Chitooligosaccharides-combination fortified noodles ameliorated central obesity and improved body composition compared to placebo.**

(A-M) Box Plots of Primary Outcome Measures at Four Time Points.

**P* < 0.05, ****P* < 0.001.

| A | 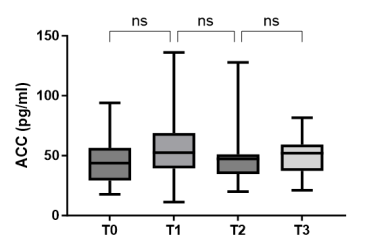 | B | 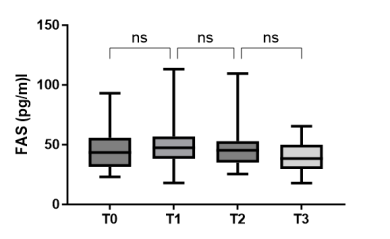 | C | 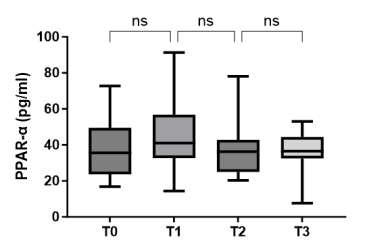 |
| --- | --- | --- | --- | --- | --- |
| D | 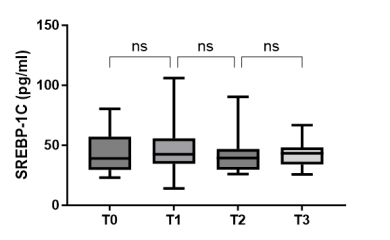 | E | 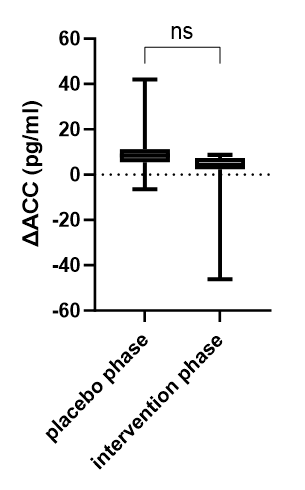 | F | 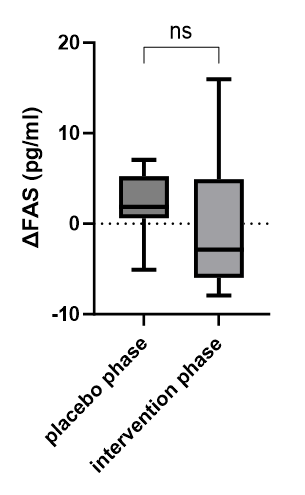 |
| G | 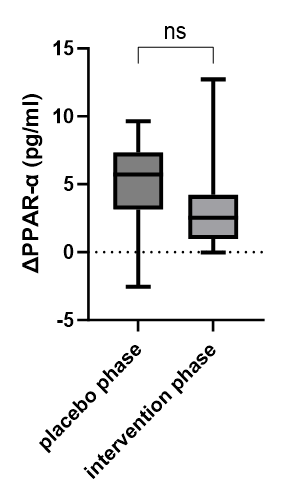 | H | 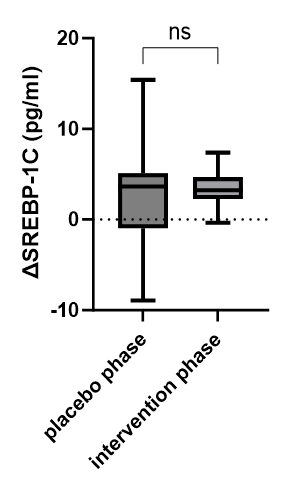 |  |  |

**Figure S2. Chitooligosaccharides-combination fortified noodles ameliorated central obesity and improved body composition compared to placebo.**

Box plots showing serum concentrations of ACC (A), FAS (B), PPAR-α (C), and SREBP-1C (D) across the four study time points. Comparison of the changes (Δ) in ACC (E), FAS (F), PPAR-α (G), and SREBP-1C (H) between the placebo phase and the intervention phase.

| A | 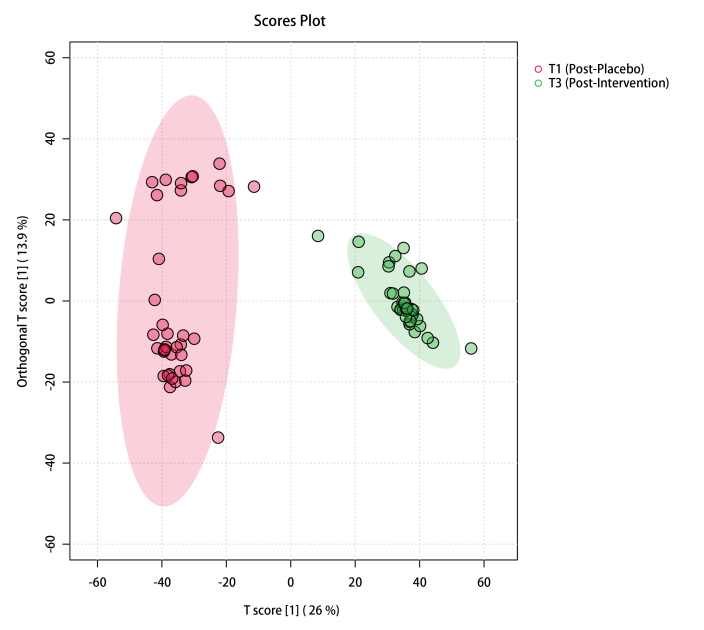 | B | 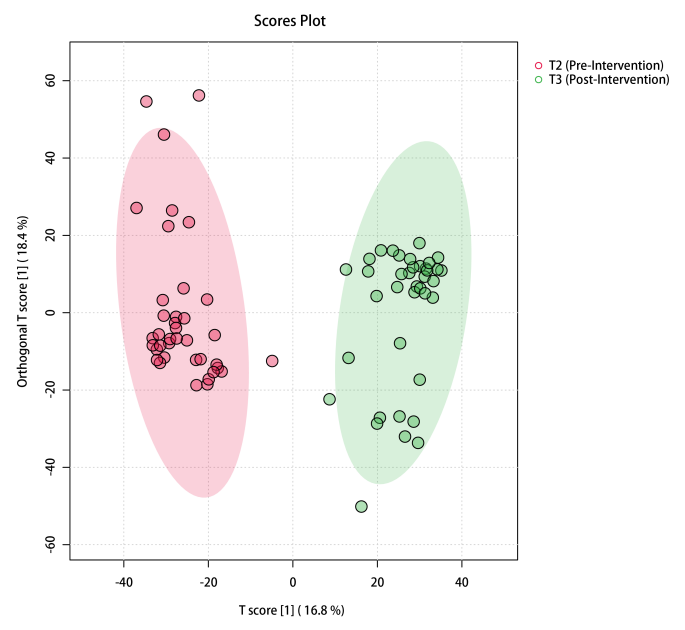 |
| --- | --- | --- | --- |

**Figure S3. OPLS-DA score plots of serum metabolomics.**

(A) T1 (post-placebo) vs. T3 (post-intervention). (B) T2 (pre-intervention) vs. T3 (post-intervention).

| A | 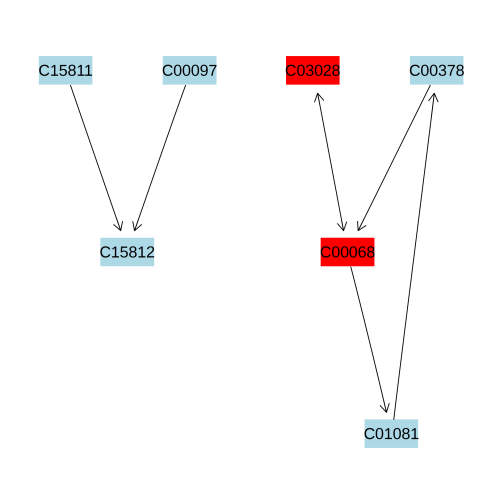 | B | 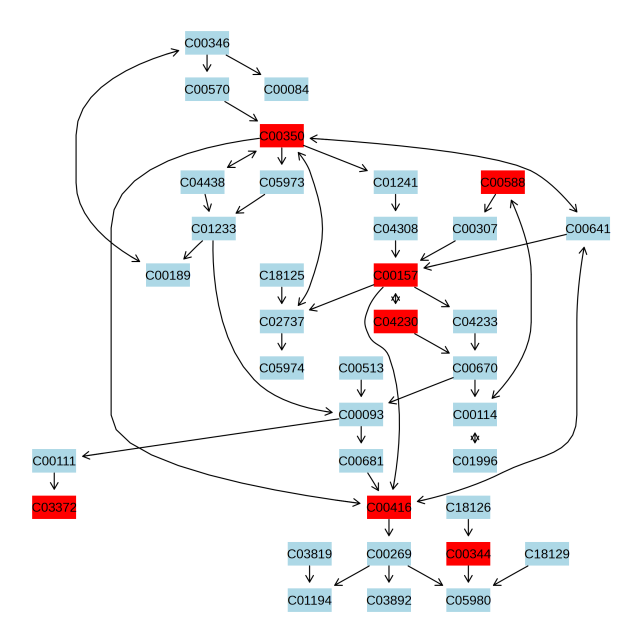 |
| --- | --- | --- | --- |

**Figure S4. Integration of Metabolomic and Clinical Phenotypes Reveals Key Metabolic-Clinical Associations.**

Enriched Thiamine (A) and Glycerophospholipid (B) Metabolism Pathway Networks. Metabolites are displayed as rectangles annotated with KEGG compound IDs. Significantly altered metabolites are highlighted in red, and non-differential metabolites are in blue. Generated using MetaboAnalyst.

| Table S1. Nutritional composition of placebo and Chitooligosaccharides-combination fortified noodles | | |
| --- | --- | --- |
| Component | Placebo Noodles | Chitooligosaccharides-combination fortified noodles |
| Per 100 g |  |  |
| Energy (kj) | 1529 | 1400 |
| Protein (g) | 11.5 | 11.9 |
| Fat (g) | 1.4 | 1.1 |
| Carbohydrates (g) | 75.4 | 74 |
| Sodium (mg) | 50 | 5 |
| Dietary Fiber (g) | 1 | 3.3 |
| Per 80 g serving (added oligosaccharides) |  |  |
| Chitooligosaccharides (g) | - | 0.4 |
| Mannose oligosaccharides (g) | - | 0.2 |
| Fructo-oligosaccharide (g) | - | 4 |

| Table S2. Baseline characteristics of land-based controls and offshore seafarers. | | | | |
| --- | --- | --- | --- | --- |
| Characteristic | Total (N=221) | Land-based group(N=148) | Offshore group(N=73) | *p value* |
| **Demographics​** |  |  |  |  |
| Age (years) | 25.00 (23.00, 28.00) | 25.00 (23.00, 29.00) | 24.00 (23.00, 26.00) | 0.138 |
| Sex, n (%) | 74 (100) Male |  |  |  |
| Length of service (years) | 6.29±5.08 | 6.66±5.22 | 5.55±4.74 | 0.128 |
| **Anthropometrics​** |  |  |  |  |
| Height (m) | 1.75 ± 0.05 | 1.75 ± 0.06 | 1.75 ± 0.05 | 0.674 |
| Body weight (kg) | 74.86 ± 9.89 | 75.08 ± 9.41 | 74.42 ± 10.87 | 0.644 |
| Body mass index (kg/m²) | 24.39 ± 2.69 | 24.44 ± 2.54 | 24.28 ± 2.98 | 0.683 |
| Waist circumference , WC (cm) | 80.21 ± 6.63 | 79.93 ± 6.10 | 80.79 ± 7.60 | 0.400 |
| **Body Composition​(by BIA)​** |  |  |  |  |
| Body fat mass (kg) | 16.62 ± 5.72 | 15.95 ± 5.69 | 17.99 ± 5.57 | 0.012 |
| Body fat percentage (%) | 21.82 ± 5.52 | 20.86 ± 5.56 | 23.76 ± 4.93 | <0.001 |
| Visceral fat area, VFA (cm²) | 69.34 ± 26.74 | 66.68 ± 27.20 | 74.71 ± 25.08 | 0.035 |
| Skeletal muscle mass, SMM (kg) | 32.78 ± 3.94 | 33.36 ± 3.71 | 31.59 ± 4.14 | 0.002 |
| Skeletal muscle index,SMI (kg/m²) | 8.31 ± 0.68 | 8.37 ± 0.63 | 8.18 ± 0.76 | 0.057 |
| Upper arm muscle circumference, AMC (cm) | 26.87 ± 1.73 | 27.03 ± 1.63 | 26.53 ± 1.87 | 0.039 |
| Bone mineral content (kg) | 3.22 ± 0.43 | 3.28 ± 0.41 | 3.10 ± 0.43 | 0.004 |
| **Serum Biochemical Parameters​** |  |  |  |  |
| Serum total cholesterol, TC (mmol/L) | 4.34 ± 0.74 | 4.28 ± 0.68 | 4.47 ± 0.83 | 0.110 |
| Serum Triglyceride, TG (mmol/L) | 0.96 (0.70, 1.32) | 0.99 (0.74, 1.38) | 0.85 (0.61, 1.28) | 0.092 |
| Serum low-density lipoprotein cholesterol, LDL-C (mmol/L) | 2.52 ± 0.65 | 2.43 ± 0.61 | 2.71 ± 0.69 | 0.002 |
| Serum high-density lipoprotein cholesterol, HDL-C (mmol/L) | 1.32 ± 0.31 | 1.34 ± 0.29 | 1.30 ± 0.34 | 0.399 |
| Serum albumin (g/L) | 47.43 ± 2.07 | 47.40 ± 2.14 | 47.49 ± 1.92 | 0.754 |
| Diastolic blood pressure (mmHg) | 75.53 ± 8.30 | 76.11 ± 8.19 | 74.34 ± 8.45 | 0.136 |
| Systolic blood pressure (mmHg) | 122.79 ± 11.41 | 125.46 ± 11.07 | 117.37 ± 10.18 | <0.001 |
| Glycated hemoglobin, HbA1c (%) | 5.36 ± 0.27 | 5.34 ± 0.24 | 5.41 ± 0.31 | 0.068 |
| High-sensitivity C-reactive protein, hs-CRP (mg/L) | 0.25(0.25,0.90) | 0.25(0.25,0.90) | 0.50(0.25,0.80) | 0.745 |
| Serum creatinine (μmol/L) | 82.96±8.55 | 83.24±8.93 | 82.40±7.74 | 0.49 |
| Urea (mmol/L) | 5.76 ± 1.12 | 5.70 ± 1.08 | 5.87 ± 1.21 | 0.290 |
| **Dietary Intake (24-hour recall)​** |  |  |  |  |
| Energy intake (kcal/day) | 2652.77 ± 712.06 | 2444.17 ± 402.78 | 3075.67 ± 973.27 | <0.001 |
| Fiber intake (g/day) | 15.35 ± 10.96 | 12.38 ± 4.66 | 21.37 ± 16.37 | <0.001 |
| **Physical Activity​** |  |  |  |  |
| Total physical activity (MET-min/week) | 3306.00 (1752.00, 5310.00) | 3586.50 (2160.00, 5959.50) | 2279.00 (693.00, 3980.00) | <0.001 |

| Table S3.Baseline Characteristics of Participants Included vs. Excluded from Metabolomics Analysis | | | |
| --- | --- | --- | --- |
| Characteristic | Metabolomics Group (n=38) | Non-Metabolomics Group (n=35) | *p value* |
| Age (years) | 24.00（23.00，25.75） | 24.00（23.00，25.50） | 0.491 |
| Height (m) | 1.75 ± 0.05 | 1.75 ± 0.05 | 0.580 |
| Body weight (kg) | 74.70 ± 11.91 | 74.13 ± 9.79 | 0.823 |
| BMI（kg/m²） | 24.43 ± 3.18 | 24.12 ± 2.79 | 0.651 |
| Waist circumference, WC (cm) | 81.13 ± 7.90 | 80.60 ± 7.37 | 0.767 |
| Skeletal muscle mass, SMM (kg) | 31.54 ± 4.65 | 31.65 ± 3.57 | 0.913 |
| Body fat mass (kg) | 18.43 ± 5.63 | 17.52 ± 5.55 | 0.488 |
| Body fat percentage (%) | 24.24 ± 4.65 | 23.25 ± 5.24 | 0.397 |
| Bone mineral content (kg) | 3.08 ± 0.48 | 3.12 ± 0.39 | 0.668 |
| Upper arm muscle circumference, AMC (cm) | 26.65 ± 2.05 | 26.39 ± 1.68 | 0.548 |
| Visceral fat area, VFA (cm²) | 74.88 ± 20.78 | 71.89 ± 27.52 | 0.610 |
| Skeletal muscle index,SMI (kg/m²) | 8.17 ± 0.82 | 8.19 ± 0.70 | 0.899 |
| Total physical activity (MET-min/week) | 2340.00（699.75，4004.75） | 2279.00（643.50，4479.00） | 0.943 |
| Serum albumin (g/L) | 47.18 ± 1.73 | 47.83 ± 2.08 | 0.148 |
| Serum total cholesterol, TC (mmol/L) | 4.61 ± 0.85 | 4.31 ± 0.80 | 0.124 |
| Serum Triglyceride, TG (mmol/L) | 0.88（0.61，1.33） | 0.77（0.67，1.23） | 0.991 |
| Serum high-density lipoprotein cholesterol, HDL-C (mmol/L) | 1.30 ± 0.35 | 1.29 ± 0.33 | 0.902 |
| Serum low-density lipoprotein cholesterol, LDL-C (mmol/L) | 2.82 ± 0.72 | 2.59 ± 0.65 | 0.158 |
